# My Experience as an Ebola patient

**DOI:** 10.4269/ajtmh.14-0763

**Published:** 2015-02-04

**Authors:** Adaora K. Igonoh

**Affiliations:** Lagos, Nigeria

I was one of the doctors in the medical team that treated the Liberian index case of Ebola virus disease (EVD) after he flew to Lagos from Liberia on the 20th of July 2014. I am not exactly sure when I came into contact with the virus. It may have been on the night (before we ever suspected Ebola) when I touched his intravenous fluid bag with my bare right hand and probably touched my eyes, mouth, or nose before I washed my hand. I worked at a private medical center, which is one of the best in Lagos. As soon as we began to suspect that the patient had EVD, we immediately notified the relevant Nigerian authorities.

Our patient denied any contact with someone with EVD or attending a funeral ceremony before illness. He had the intention of attending a conference in a different Nigerian state and wanted at all cost to be discharged, even if against medical advice. His conditioned worsened very rapidly, with persistent fever, vomiting, diarrhea, and dehydration despite aggressive intravenous rehydration. He manifested hemorrhagic symptoms, including hematuria and subconjunctival ejection. His breathing was labored and he was confused. I found him dead on the toilet seat 5 days after admission.

After the possible exposure to Ebola virus, all hospital staff were placed under surveillance. Each person was given a temperature chart and thermometer, asked to measure body temperature morning and night for 21 days, and instructed to call an Ebola Helpline at the first sign of a fever.

This was the scariest time of my life. I could not get over my anxiety. I would wake up in the middle of the night just to check my temperature. Eight days after my first contact with the index case and 4 days after he died I began to feel unwell, with joint and muscle aches. It felt like malaria so I treated myself with anti-malarials. Two days after the onset of the first symptoms, I developed a fever. My sore throat progressed gradually. I quickly developed a loss of appetite, and I felt absent from my environment.

I noted every symptom and sign, but I did not realize how quickly they had progressed. It did not initially register in my head that I had EVD. Like many people, I was in denial at first. Nobody wants to be diagnosed with EVD. Even many educated patients do not believe they have the disease until the confirmatory test result is received. We often choose to believe it is some other disease that we can handle—certainly not one with a mortality rate up to 90%.

On the second day of fever, I called the Ebola Helpline and an ambulance came for me within a few hours. Blood samples were taken, the EVD result was positive, and I was taken into isolation. This result was devastating. The constant news about Ebola on television and radio were not helping matters at all.

The first person I met when I got to the isolation ward was a World Health Organization (WHO) doctor, Dr. David Brett-Major. He was the only doctor at the time and he came in to see us at least twice daily, shuttling between the male and female wards. He helped out with cleaning up as well, because the nurses were too scared to enter the ward. He told me that 90% of my response to the infection was dependent on me, as there was no specific antiviral treatment, only supportive therapy.

The major concern was treating and preventing dehydration from fluid loss. Dr. Brett-Major helped mix my first bottle of oral rehydration solution (ORS) and challenged me to drink at least 4.5 L of the fluid daily. He would spend time chatting with us individually, and I could tell he was a cheerful person even though the only part of his face I could see was his eyes through his misty goggles. I was impressed and touched that he came all the way from Geneva to care for us. Soon after, volunteer doctors started to show up at the ward, running about three shifts daily. I recognized some of them and it made me comfortable being in the hands of people I knew and had worked with. They knew we were lonely, understood our fears, and always tried to put smiles on our faces. The heat was overwhelming. I couldn't imagine wearing the personal protective equipment (PPE) for more than 5 minutes. Emergency care was not at its best because it took a long time for the doctor to put on fresh PPE and get into the ward to see a patient. If a patient was on intravenous fluids, someone was needed to ensure that a new bag of fluids was hung. Because they were short staffed, I did most of the work managing intravenous lines for critical patients who could not consume ORS.

Notable among the volunteer doctors was one of my consultants during internship at the Lagos University teaching hospital and a resident doctor I had worked with. I remain grateful to these heroes for going where so few dared to go, putting their lives at risk to help to save us.

Some of the volunteers ran away after a few days. I understand; it could not have been easy staring death in the face. There were others who volunteered to work in the wards, showed up, but evaded the responsibility conferred on them to help the sick. They would be around, but would not respond when called by patients.

I was kept in a room that stank of depression. Indeed, depression had become the order of the day in the ward, with patients suddenly crying out of frustration. There was no form of entertainment. We had so many unanswered questions. Were we going to survive? How long would we be in the ward? Why did some of us seem to get better, only to suddenly deteriorate and succumb to the infection? Who exactly were our caregivers, all covered up in masks and suits? It felt like I was a dangerous alien from whom people needed protection.

I battled for my life while watching patients die one by one next to me. Was it my turn? I could not accept that. The fever was weakening me. The constant diarrhea was degrading enough to make me wear adult diapers. The vomitus was so bitter that it made me want to vomit even more. Sleeping was so difficult because no position was good enough for my severely aching joints. There was a heaviness in my chest that made it hard to breathe. I woke up most mornings to sharp pain in my eyes from the sunlight, my first experience with photophobia. My body was soon covered with red spots, particularly my arms and legs.

We EVD patients all noticed that we were urinating more than usual. I woke up several times a night to pass urine, which was foamy and concentrated. I knew it was not as a result of the many bottles of ORS that I consumed because I still felt dehydrated and my throat felt dry. This phenomenon reduced as my symptoms subsided. I observed that some of my fellow patients had a zombie look, very aloof and a little scary, as though they were not present in their bodies.

The only treatments I received were bottles of ORS, paracetamol and vitamin supplements. I drank like my life depended on it. I prayed to God like my life depended on it. Five days after my admission, when I thought I could take no more, the vomiting started to subside. I ate a lot of bananas because I suspected my potassium level was quite low. Soon after, the diarrhea stopped and the fever subsided, only to return later, due apparently to malaria from the legion of mosquitoes that shared the room with us. Based only on a hunch, because routine malaria testing was not performed as a result of safety concerns, I decided to self-treat for malaria. The fever was gone on the second day of treatment. I was discharged 14 days after I was admitted after blood tests for Ebola virus came back negative. That gave other patients hope that they would one day leave the ward too.

Recovery has been gradual, but progressive. My appetite returned to what it was like pre-EVD after 6 weeks. Fatigue lingered on for months, but gradually improved. I lost about two-thirds of my lovely hair and had to have it all shaved. I struggled with fleeting joint pains for months. The joints would suddenly become painful and swollen. After a few days the pain would disappear, only to reappear in another joint days later. Now, three and a half months after leaving isolation, I feel much better and am ready to pick up from where I left off.

Fortunately, the swift response of the Lagos Ministry of Health to contain the outbreak was successful. I have learned that with the right strategies, tools, and resources, we can stop this epidemic. Containing an outbreak is a collaborative effort. Every arm of government must be involved to mobilize all resources available to fight our common enemy. When a case is confirmed, the government must act swiftly to stop the chain of transmission. The right information must be disseminated to control fear and panic and prevent stigmatization. Some traditional rulers informed their people that the consumption of salt and water at a certain time of the day would help prevent and cure EVD. This unfortunately led to the death of a number of people who diligently adhered to the advice.

What have I learned from my experience with EVD? First, volunteer healthcare workers treating EVD patients, in addition to administering drugs, should understand that a smile, a hand shake, and words of encouragement greatly help to lift the spirits of patients. Second, hope is necessary for survival. Videos of interviews with survivors would be valuable, teaching patients that their diagnosis is not a death sentence. Third, the health workers needed to take short shifts because it was difficult staying in the PPE for more than 30 minutes. A fatigued healthcare worker is a risk to the patient, to him or herself, and to the public. Thus, caring for EVD patients requires a great deal of manpower to appropriately manage complex patients and avoid the fatigue that can lead to breaches in protocol and possible healthcare worker infection.

I remember the ones we lost in this battle against Ebola: my mentor, Dr. Adadevoh, the Consultant Physician who did not let the index case leave the hospital despite threats, Nurse Justina Ejelonu who contracted the virus on her first day of work while tending to the index case, and the other brave men and women who paid the ultimate price to save others. They are the real heroes we can never forget. It is my hope that we see ourselves as connected to each other. The world is a global village and we must be our brothers' keepers. Let us work together to fight and conquer Ebola.

